# Editorial: Enterobacteriaceae antimicrobial agents and resistance: relationship with the therapeutic approach, volume II

**DOI:** 10.3389/fcimb.2024.1356413

**Published:** 2024-01-18

**Authors:** Maria Teresa Mascellino, Silpak Biswas, Alessandra Oliva

**Affiliations:** ^1^ Department of Public Health and Infectious Disease, Sapienza University, Rome, Italy; ^2^ Department of Microbiology, School of Tropical Medicine, Kolkata, India

**Keywords:** multi-drug resistant *Enterobacteriaceae*, innovative therapies, association of antibiotics, plasmids, humoral immunity

Antimicrobial resistance (AMR) is one of the major global health issues worldwide (Hu et al., 2016). The situation is becoming increasingly drastic, especially in the last years, due to the increase of new antibacterial agents that, in the long run, lose their effectiveness, becoming useless or even harmful for infections treatments, thereby leading to a greater resistance selection (Laxminarayan et al., 2016). Consequently, antibiotics become ineffective, increasing the risk of disease spread, severe illness, and death (Palacios-Baena et al., 2021). New strategies should be taken into account in the therapeutic approach through enhancing host immunity mechanisms or relying on new compounds, such as essential oils (Oliva et al., 2018), N-acetylcysteine (Oliva et al., 2021), bacteriophages (Qin et al., 2021), vaccines (Diago-Navarro et al., 2017), or combination therapy (Mascellino et al., 2021) (**Figure 1**).

**Figure 1:**
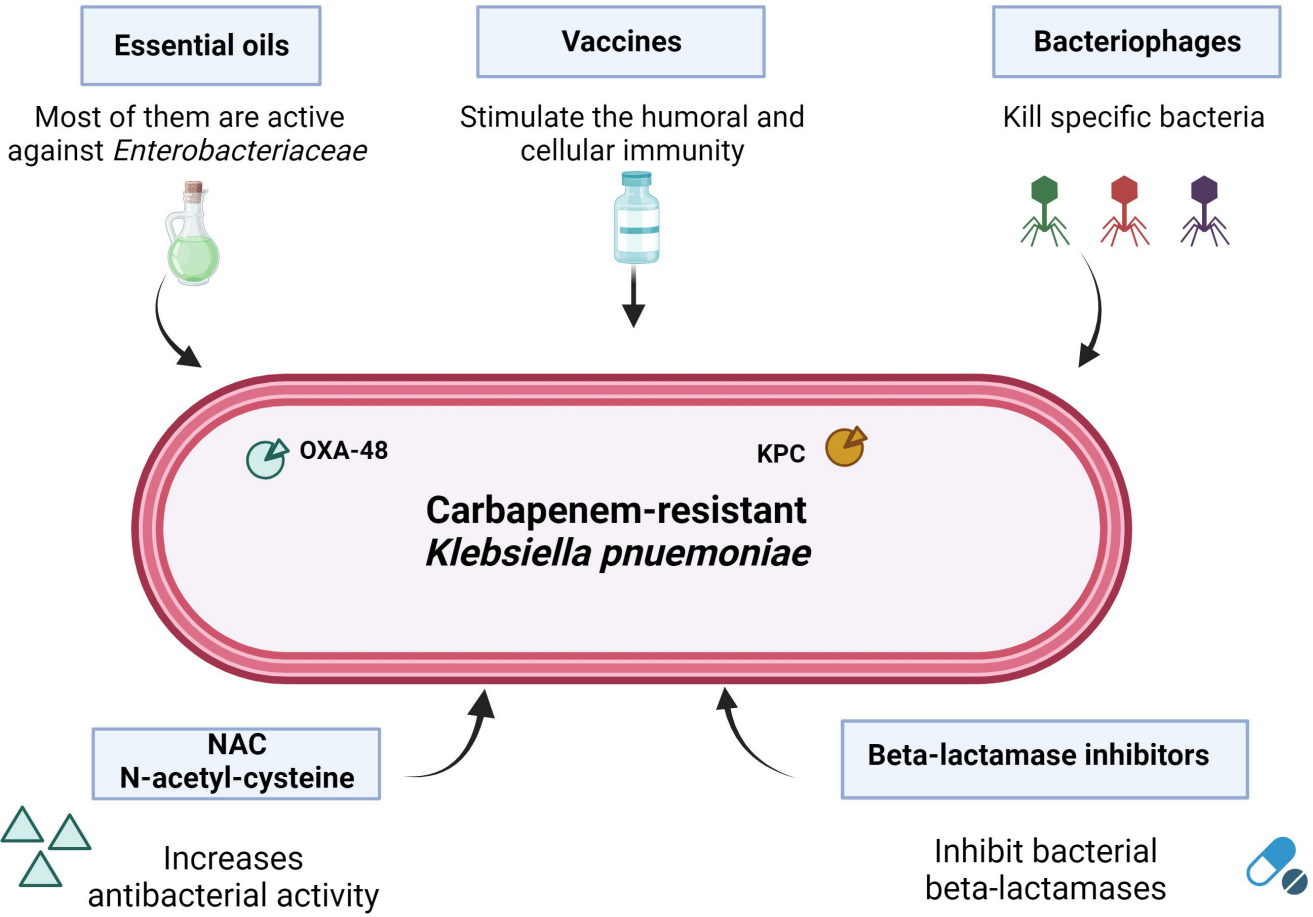
Activity of different compounds against resistant *Klebsiella pneumoniae* (figure made by Biorender.com; accessed on December 15).

The presence of enzymes, such as extended-spectrum β-lactamase (ESBL) and carbapenemases (KPCs, Metallo β-lactamases, and OXA), constitutes the principal resistance mechanism to antibiotics (Scudeller et al., 2021).

A new metallo-β-lactamase called SZM-1 was identified and characterized in Shenzhen Bay, South China, by Fang et al.

This Shenzhen metallo-β-lactamase, belonging to an *Arenimonas* metagenome-assembled genome recovered from the river sediment in the Shenzhen Bay area, shows carbapenemase activity and confers an increasing resistance toward carbapenems in the carrier bacteria.

In order to test the most common resistance genes (*blaKPC*, *blaNDM-1, blaIMP-1 group, and blaVIM*), Kim et al. developed a novel method, loop-mediated isothermal amplification (LAMP)-based assay using eight reference Gram-negative bacterial strains. The results of the LAMP assay were compared to those of conventional PCR and appeared quite similar, with a good specificity and sensitivity, thus making this method appropriate for detecting the above four β-lactamase genes. The low complexity and low cost of this assay may be suitable for most types of laboratories and may be beneficial for low-income countries.

Nosocomial infections by multidrug-resistant (MDR) organisms are among the main causes of morbidity and mortality in patients hospitalized in intensive care units (ICUs). For this purpose, the study of Li et al., which deals with the less frequent bacterium of *Enterobacter bugandensis*, is useful, because this microorganism is mainly involved in nosocomial infections, and MDR strains have been isolated from various environments. This emerging pathogen causes serious problems in nosocomial infections treatment. Among the various mechanisms of antimicrobial resistance, active efflux pumps are a well-known system that eliminates clinically relevant antimicrobial agents, rendering specific pathogens resistant to the activity of multiple drugs. The authors studied the activity of TolC, an outer membrane component of different efflux pumps, in respect to the susceptibility to a range of 26 antimicrobial agents in *E. bugandensis* strains, leading to excellent results that can be used as a reference for the future emergence of MDR isolates belonging to this species.

Two interesting articles based on the association of β-lactams together with β-lactamase inhibitor activity are included in this Research Topic. The first one by Kang et al. evaluated the efficacy of ceftazidime–avibactam and aztreonam–avibactam against bloodstream infections or lower respiratory tract infections caused by extensive drug-resistant or pan-drug-resistant *Pseudomonas aeruginosa*. Both associations turned out to be effective against this microorganism. The second article by Zhang et al. examined the activity of piperacillin–tazobactam (PTZ) compared to meropenem (MER), which is commonly used to treat complicated urinary tract infections. Both MER and PTZ were active (≈ 80% eradication rate); however, the adverse events were found to be more common in MER than in PTZ.

*Klebsiella pneumoniae, Acinetobacter baumannii*, and *Pseudomonas aeruginosa* appear to be the most commonly implied microorganisms in antimicrobial resistance. Carbapenem-resistant *Klebsiella pneumoniae* is detected frequently in blood-stream infections, causing high mortality rates especially in ICUs, and in hospital-acquired infections (Li et al.).

In the same way, the presence of carbapenem-resistant *Acinetobacter baumannii* (CRAB) is very common, as reported by Huang et al. in China. CRAB is highly refractory to conventional clinical antibiotics, with the exception of cefoperazone/sulbactam. Particularly, the presence of OXA-23 and OXA-51 resistance genes is common in these strains, and together with TEM β -lactamase, they are most the dominantly involved factors making the multidrug-resistant CRAB the main pathogen of nosocomial infections all over the world.

The articles of Shang et al. and Hadjadj et al. describe the colonization and outbreak of resistant germs.

In the first one, it was noticed that extended-spectrum β-lactamase-producing *Enterobacteriaceae* colonization does not interfere, following infections by ESBL, with either ESBL Gram-negative bacteria or liver transplant outcomes. Consequently, this colonization cannot be considered a risk factor for possible post-transplant Gram-negative infections, even if some previous literature data provide conflicting results mainly for Gram-positive bacteria (Russell et al., 2008).

In the second article, the outbreak of carbapenem-resistant *Enterobacteriaceae i*n a thoracic oncology unit was taken into consideration. The study included *Citrobacter freundii* and *E. hormaechei* (both belonging to the *Enterobacter cloacae complex)* producing OXA-48 carbapenemase in hospitalized patients. The *blaOXA-48* gene was disseminated by both clonal and plasmid spread. The origin of this outbreak appears to have been both external and internal to the ward, meaning that this cross-infection had to be managed with the implementation of appropriate prevention and control measures.

Lastly, an effective new strategy for fighting antimicrobial resistance (AMR) could rely on increasing host immunity by making people less receptive to infections and more ready to combat them. This interesting topic has been elucidated by Liang et al. This article has demonstrated that host defense plays a critical role in killing *K. pneumoniae* and, hence, sheds light on the mechanisms underlying bacterial immunity, such as mechanical barriers, innate immune cells, and cellular and humoral immunity, in order to provide new strategies for the clinical treatment of *K. pneumoniae* infection. Understanding host immune mechanisms would facilitate the comprehension of the pathogenesis and could provide new ideas for future treatment of this infection in the era of antibiotics.

In conclusion, AMR is currently a crucial issue in the management of infectious diseases worldwide (Jamal et al., 2020). New antibiotics, antimicrobial associations, and combinations between beta-lactams and beta-lactamases inhibitors are taken into account, even if bacterial resistance and the therapy failures are always lurking (Bhatnagar et al., 2021). Appropriate prevention and control measures should be greatly improved (van Seventer and Hochberg, 2017). This Research Topic addresses an increasing problem that requires drastic solutions for limiting the further spread of resistant bacteria. Alternative therapeutic programs could be proposed, such as using N-acetyl-cysteine, bacteriophages, and, more importantly vaccines, stimulating the immune response that can recognize different bacterial antigenic epitopes in order to promote pathogen elimination.

## Author contributions

MM: Conceptualization, Supervision, Writing – original draft, Writing – review & editing. AO: Data curation, Investigation, Methodology, Writing – review & editing. SB: Formal analysis, Visualization, Writing – review & editing.
